# A Woman's Clothes-Philosophy

**Published:** 1889-05-11

**Authors:** Xantippe Teufelsdröckh


### A Woman's Clothes-Philosophy.

By Xantippe Teufelsdröckh.

The University of Weissnichtwo having established a chair of clothes-philosophy as a memorial of my late relative, Herr Diogenes Teufelsdröckh, did me the honour to elect me to the professorship thus created. My appointment is due, I think, to two considerations: First, that sense of courteous regard for the honoured dead which determines the result of so many elections; and secondly, that feeling that whatever a man can do well a woman can do better, which is one of the chief articles in the creed of progress. Without venturing an opinion on this latter point, I may say that I feel that the question of clothes comes directly within my province, and that I con with the utmost care, and think over with the deepest interest, all the literature of the subject.

Among other things, I have lately received a pamphlet on "Clothing," by Francis Vacher, F.R.C.S., medical officer of health for Birkenhead. Everyone who feels deeply on the subject of the garments which we wear, and which, whether we mean it or no, express so much of our individuality, must agree with the general principles Dr. Vacher lays down. Whether we look at wearing apparel from the hygienic standpoint implied in the word "clothing," or the æsthetic aspect indicated by the term "dress," we will agree that it should be a bad conductor of heat, yet should not be harsh or irritating; should wash and wear well; should be suited to the season and climate, and to the age and occupation of the wearer. It is satisfactory to note, too, that Dr. Vacher does not preach the cult of Jaegerism to its farthest extreme. Woollen underclothing is undeniably desirable, as tending to maintain an equable heat of the body under considerable variations of surrounding temperature; but one of its functions is *not*, as has been asserted, to irritate the skin in order to maintain a constant blood supply, and to scrape off the epidermis. The circulation of a healthy person does not need this continual titillation, and at night especially it is undesirable. All necessary movement of the blood will go on without being flogged by these little whips of flannel fibre, and for the circulation, as for the organism at large, comparative inactivity during sleep is best for health. When warmth is desired, the soft "flannelette"—now so cheap—forms an admirable material for nightdresses, though it must be admitted that it does not stand what the Greenlander called "too much washum."

It is consoling, also, to notice that Dr. Vacher defines one of the minor functions of dress to be the concealment of defects of form, due to age, scars, or any other cause; and frankly says that to this end a little padding is allowable. But I, as a woman, begin to quarrel a little with Dr. Vacher when I see that he allows the little artifices practised by man, and finds fault with those indulged in by women. Men pad their coats, so padding is permissible; but they do not put whalebone in the seams, therefore the stiffening of dress bodices by this means is forbidden. Now, I appeal to every woman to bear witness that the function of these bones or steels is not to compress the figure, but to prevent the bodice from "rucking up" into ungainly folds, the more decided curves of the feminine figure making such a thing more likely to happen to their clothes than to men's. On the whole, Dr. Vacher favours his own sex too much. "It is scarcely beyond the truth," he tells us, "to say that men have attained to something near perfection as regards cloth clothes." Is this so? If it is—well, I can but comfort myself by the remembrance that we women have always been held the more charming for our very imperfections.

Among our imperfections is to be placed the skirt. Dr. Vacher evidently approves of the "dual garment" for women, and uses that strange plea of its advocates—that when it is on you wouldn't know the divided skirt from the undivided one. Practically, the plea amounts to this—you may encase your legs in two loose and much-frilled petticoats instead of one. They will catch the mud as much as the old garb; they will present but little difference from the ordinary dress; but you will have the satisfaction of knowing that you are wearing a divided skirt, and that nobody would guess it. Is this worth striving for? Most women now wear woollen under-garments of the most approved type, and the sanitary dress reformers deserve full credit for having taught that a few warm, fitting garments are better than many loose ones; but for the outer garment it is very certain that the skirt has a long future before it. It should be useless to point out to a medical man that reason why the petticoat has been, and will continue to be, the dress of civilised woman, and why, in all probability, it will always have cunning folds breaking its lines here and there. There is an apology to be made even for the crinoline.

Naturally, the corset is condemned, and the old, old tale is repeated—that it compresses the waist into an unnatural circular shape. Now, as the waist of a corset itself is not round, it is difficult to say why it should have that effect; and, as a matter of fact, the waists of very few women approach the circular form. The same plea may be used for the corset as for the skirt. For young, slim women it is needless; they can do without it, just as they could wear the divided skirt, even though it approached more closely to the bathing-costume ideal, with grace and fitness. But their mothers, who have lost delicacy of outline, and have grown stout with years, claim the support of the corset; and, in all probability, they will continue to get it. This is one reason why the corset still obtains. Another is the approval of man. For a sex-instinct, too deeply founded for any argument to destroy, makes woman desire man's admiration. When you want to conquer a man, set a woman to smile on him; but when you seek to win a woman to any doctrine under the sun, give her a man for teacher. And, as yet, men like slim waists in women. "They're very unhealthy, I suppose," a young doctor said to me, not long ago; "but"—with a sigh—"they're so pretty." It is said, however, that the recent marriage of a duke who disapproved of corsets, and chose his bride accordingly, has made for the emancipation of women in that matter. Let us devote our attention to the training of our dukes, and the corset will vanish from the land.

But, more than is generally admitted, convenience guides fashion. The chief objection to the so-called æsthetic dress was that though graceful enough when the wearer was standing or lounging in a luxurious attitude, it was not suited for the quick movements of a busy woman. In the drawing-room it was well enough, but it was not suited for streets, trains, and tramway cars. On other points a truer artistic instinct than is generally conceded to women, guides our choice. Why do girls wear hats and older women bonnets? I will venture a surmise. In youth a woman's chief charm lies in her bright eyes and rosy cheeks, which shine out more clearly and bewitchingly from under the shade of the hat. But as years go on and the eyes grow dim, and the complexion fades under the cares, the duties, and the sacred burdens of womanhood, there comes instead a sweet gravity, a majesty, on the forehead, even when its smoothness is marred by wrinkles, that one loves to look upon, and that the bonnet shows off best.

I have enumerated some of the points on which I differ from Dr. Vacher, because I think he, in common with most lecturers on dress, fails to grasp the reason why women cling so persistently to things which are so frequently and, I think, so thoughtlessly condemned. But it remains to be said that his principles are sound, and the reasons given for them such as appeal to the common-sense of all.

